# Miscellany

**Published:** 1888-02

**Authors:** 


### Miscellany.

---

For Washing out the Bladder, Ultzmann, of Vienna, recommends, in *Centralblatt für Chirurgie*, the following: For irritable bladder, lukewarm water, with a little tincture of opium, or solution of cocaine, ¼ per cent.; or resorcin, ½ per cent.; or carbolic acid, ⅙ per cent. Solution of permanganate of potassium, $\frac{1}{10}$ per cent.; or amyl nitrite, gtt. iij, to water, Oj, will prevent decomposition of the urine. He recommends for phosphaturia $\frac{1}{10}$ per cent. salicylic acid.

---

The *St. Louis Weekly Medical Review* says that a young Texas physician, called to his first case of labor, found the bag of waters presenting, which, mistaking for the bladder, he tried to replace, with the result of rupturing it, allowing the fluid to escape. Rushing frantically from the room to a neighboring physician, he cried: "By Jove! she's busted. Get your instruments—she won't live an hour."

An Examination for License to Practice.—The Board of Health of Dakota recently examined an application for a license to practice medicine. He had been practicing medicine for years in Dakota. Here are some questions and answers: "What medical paper do you take, Doctor?" "Well, I can get along without them." "What books have you in your library?" "'Gunn's Family Physician and Common-Sense Home Doctor.'" "Name the three great cavities of the body?" "The head, the belly, and the diaphragm." "Name contents of abdominal cavity?" "Kidneys and the prostate gland." "Have you treated any cases of enlarged prostate?" "Lots of them." "With what success?" "Tiptop! never lost a case." "Did you ever treat any female for enlarged prostate?" "Oh, yes; numbers of them."

---

Another Cure for Consumption.—Prof. Luton, of Rheimes, asserts that a cure of tuberculosis can always be effected by the employment of the phosphate of copper, which, however, must be in the nascent state and soluble in an alkaline body. His formula is as follows:

| | | |
|---|---|---|
| ℞—Acetate of copper neutral . . . . . . | grammes | 0.15 |
| Crystallized phosphate of sodium . . . | grammes | 0.75 |
| Glycerine and powdered licorice, of each, | | q. s. |

This for one pill.

---

The Wonders of the Telephone.—A physician reports to us, says the *Medical Age*, December 10th, that he was saved a two-mile ride through a driving storm the other night by having the patient, a child, brought to the instrument and held there until it coughed. He diagnosed false croup, prescribed two grains of turpeth mineral, and turned in for an undisturbed sleep during the remainder of the night. He found the patient in the morning doing nicely—under the care of another doctor.

---

Dr. Bartholow's favorite prescription for cholera infantum is:

| | |
|---|---|
| ℞—Bismuthi s. nit. . . . . . . . . . . . . . | ʒ i. |
| Pepsini sacch. . . . . . . . . . . . . . | gr xxx. |
| Zinci oxide . . . . . . . . . . . . . . | gr. v. |

Met. Ft. Chart. No. 6. Sig: One every 4 to 6 hours, or oftener if necessary.

A Physician going his rounds among some small-pox patients in a hospital, and stopping by the bedside of an Irishman, inquired: "Well, Pat, how are you to-day?" "Faith, sir, I'm better; but I am so wake that I should not be surprised at all if some one was to come and tell me I was dead."

---

The *Medical World*, Philadelphia, has issued a visiting list on a new plan. It is in sections—one book for each month. A neat leather case is provided, into which the monthly parts are slipped. By this means, plenty of space is provided, and, at the same time, a small flat book, convenient for the pocket.

---

Nutrient Enemata.—Prof. Ewald, of Berlin, has recently made some careful experiments with different nutrient enemata, and has found that enemata of eggs were of decided service, and that they were as efficient and satisfactory without being peptonized as when they were subjected to this process.

---

A Homeopathic doctor, addressing a graduating class of nurses the other day, said: "Some trained nurses are trained nuisances. The tongue is the most dangerous thing provided you by nature. Learn to keep your mouth shut." Good advice for other than homeopathic nurses.

---

The *Scientific American* suggests as a means of relief in chilblains the following: Dissolve an ounce of ammonium chloride in half a pint of cider vinegar, and apply frequently. A half-pint of alcohol may be added to this lotion with good effect.

---

Wife (to third husband)—"If you feel so unwell, John, I think we had better send for my old family physician." Third husband (somewhat hastily)—"No, my dear, I would prefer to send for some one else."

---

Antiseptic Candles are the latest novelty. They are made by combining iodine and salicylic acid and incorporating with fats, paraffine or wax. Phenol is produced by the decomposition of the salicylic acid.

---

Lawyer (in hoarse whisper)—"Doctor, I have such a cold this morning that I can't speak the truth." Doctor (sympathetically)—"I am glad it isn't anything to interfere with your business."

